# Exploring Vascular Function Biomarkers: Implications for Rehabilitation

**DOI:** 10.21470/1678-9741-2016-0085

**Published:** 2017

**Authors:** Shane A. Phillips, Daniela Kuguimoto Andaku, Renata Gonçalves Mendes, Flávia Rossi Caruso, Ramona Cabiddu, Rodrigo Boemo Jaenisch, Ross Arena, Audrey Borghi-Silva

**Affiliations:** 1 Department of Physical Therapy, Integrative Physiology Laboratory, College of Applied Health Sciences, University of Illinois at Chicago, Chicago, IL, USA.; 2 Cardiopulmonary Physiotherapy Laboratory, Department of Physiotherapy, Universidade Federal de São Carlos (UFSCAR), São Carlos, SP, Brazil.

**Keywords:** Rehabilitation, Atherosclerosis, Endothelium

## Abstract

The endothelium plays an important role in maintaining vascular homeostasis and regulating blood vessel function. Endothelial function is considered an independent predictor for risk of future cardiovascular events in cardiovascular and non-cardiovascular patients, as well as a predictor for postoperative complications in cardiovascular surgery patients. Brachial artery flow-mediated dilation by high-resolution ultrasound is widely used to evaluate endothelium-dependent vasodilation, which is mainly mediated by nitric oxide release. Physical exercise exerts beneficial effects on endothelial function and can be used in both primary and secondary prevention of cardiac and peripheral artery diseases, even in the postoperative period of cardiovascular surgery.

**Table:** 

Abbreviations, acronyms & symbols				
CAD	= Coronary artery disease		HIAE	= High intensity interval aerobic exercise
CAE	= Continuous moderate intensity aerobic exercise		IMT	= Intima-media thickness
CHS	= Cardiovascular Health Study		LVEF	= Left ventricular ejection fraction
CV	= Cardiovascular		MI	= Myocardial infarction
CVD	= Cardiovascular disease		NADH	= Nicotinamide adenine dinucleotide
CPB	= Cardiopulmonary bypass		NADPH	= Nicotinamide adenine dinucleotide phosphate
CR	= Cardiac rehabilitation		NO	= Nitric oxide
EDCF	= Endothelium-derived contracting factors		OH.	= Hydroxyl radicals
EDHF	= Endothelium-derived hyperpolarizing factor		PAD	= Peripheral arterial disease
EDRF	= Endothelium-derived relaxing factors		PCI	= Percutaneous coronary intervention
EDV	= Endothelium-dependent vasomotion		PGI_2_	= Prostaglandin I_2_
eNOS	= Endothelial NO synthase		ROS	= Reactive oxygen species
ET-1	= Endothelin-1		SOD	= Superoxide dismutase
FMD	= Flow-mediated dilation		uAP	= Unstable angina pectoris
GPx	= Glutathione peroxidase		VEGF	= Vascular endothelial growth factor
H_2_O_2_	= Hydrogen peroxide		VTI	= Velocity time integral

## INTRODUCTION

### The Role of the Endothelium in Regulating Vascular Health

The endothelium is the first line of defense of end-organs against external aggression and plays an important role in maintaining vascular homeostasis^[1]^. Endothelial cells control vascular function by responding to various hormones, neurotransmitters and vasoactive factors which affect vasomotion, thrombosis, platelet aggregation and inflammation^[2]^. The endothelium mediates the vasomotor tone of the circulation in response to various chemical (acetylcholine, ACh) or physical (shear stress) stimuli^[3]^, by synthesizing and releasing different vasodilator and vasoconstrictor mediators. There are endothelium-derived relaxing factors (EDRF), including nitric oxide (NO), prostaglandin I_2_ (PGI_2_), and endothelium-derived hyperpolarizing factor (EDHF), but also endothelium-derived contracting factors (EDCF), including endothelin-1 (ET-1) representing the most potent molecule.

### Mechanisms of Vasodilator Function

NO has been shown to play an important role in the maintenance of basal vasodilator tone of the blood vessels and plays a key role in vasodilation^[4]^. It also prevents platelet adhesion and aggregation, as well as leukocyte adhesion and migration into the arterial wall and inhibits smooth muscle cell proliferation and intimal migration, oxidation of LDL cholesterol, apoptosis of smooth muscle cells, all key events in the development of atherosclerosis^[2]^.

### Mechanism of Flow-Mediated Vasodilation

Flow-mediated dilation (FMD) is a physiologically important stimulus regulating vascular tone and homeostasis of the peripheral circulation. This important endothelial mechanism of vasodilation occurs in virtually every vascular bed. In large arteries, FMD may be critical for preventing atherosclerosis through release NO. In humans, a reduction in FMD is prognostic of cardiovascular disease (CVD). Animal studies have reported that the contribution of NO to FMD is reduced as oxidative stress increases in the presence of risk factors for CVD^[5]^. In humans, *in vivo* and *in vitro* studies have demonstrated that relaxant factor(s), other than NO, compensate to maintain FMD when NO availability is reduced^[6]^. Altered endothelium-dependent FMD is a hallmark of the development of CVD and is an initiating event in the development of atherosclerotic heart disease^[7]^. During coronary artery disease (CAD), arterioles exhibit altered endothelium-dependent vasodilation^[8]^. In humans, Phillips et al.^[6]^ have shown that hydrogen peroxide (H_2_O_2_) replaces NO as the mediator of endothelium-dependent flow-induced dilation in resistance arteries of visceral fat in the presence of CAD. An increase in oxidative stress appears to be a major mechanism underlying the development of vascular endothelial dysfunction. The dominant mechanism responsible for endothelial dysfunction is the decrease in bioavailable NO, as well as the increase in reactive oxygen species (ROS) production. The generation of ROS in the endothelium includes anions (O_2_), hydroxyl radicals (OH^.^) and hydrogen peroxide (H_2_O_2_). ROS modulate vascular tone by several mechanisms; directly act as EDCF or indirectly potentiate EDCF mediated responses by reducing the bioavailability of NO. ROS might interact with NO and reduce its bioavailability via different pathways: direct NO inactivation by superoxide with peroxynitrite (ONOO^-^) formation; reduction in NO synthase expression and activity due to changes in their substrate or cofactors, and also endothelial NOS uncoupling^[9]^.

### Methods in Evaluating Peripheral Vascular Function

#### Rationale for Measuring Peripheral Arterial Function

The endothelium occupies a unique position in that it is able to secrete a variety of vasoactive molecules and is also exposed to direct vascular injury. It is thus an important mediator of atherosclerosis formation and is widely perceived to be a metric of vascular risk. Previous studies have demonstrated a correlation between measures of coronary vasodilator function and FMD^[10]^. Early studies established that attenuated vascular responses occur prior to the development of atherosclerosis in response to a milieu of risk factors, thus making measurements attractive as a screening tool for cardiovascular (CV) risk^[7]^. Endothelial function is dynamic and can be attenuated rapidly in response to acute oxidative stress (cigarette smoking, high fat load). In addition, interventions that are associated with a decrease in vascular risk will improve vasodilation within a period of months allowing one to determine the impact of novel interventions in a timely fashion^[11]^.

### Non-invasive Conduit Vessel Endothelial Function

#### Brachial artery FMD

Celermajer et al.^[12]^ showed the first report of the measurement of peripheral artery FMD in 1992. Not only were they able to describe a new method, but also demonstrated that children with familial hypercholesterolemia had impaired function at an early age. Since then thousands of studies have been reported using this methodology. The guidelines for measuring brachial artery FMD are summarized previously^[3]^. Briefly, a high resolution (> 10 MHz) linear array ultrasound probe is used to longitudinally image the brachial (or radial) artery at rest. A thin blood pressure (BP) cuff is inflated to supra-systolic pressure for 5 minutes on either the forearm or on the upper arm. After the cuff is released, the artery dilates in response to shear stress mediated NO release and maximum dilation typically occurs between 45 and 120 seconds^[13]^. After a 5 minutes recovery period, sublingual nitroglycerine may be given to assess endothelium-independent dilation. The FMD response has been shown to be mediated mainly by NO, thus it reflects endothelium-dependent vasodilation^[14]^. Two large cohort studies, the Cardiovascular Health Study (CHS)^[15]^ and the Multi-ethnic Study of Atherosclerosis^[16]^, demonstrated that FMD was an independent predictor of CV outcomes. FMD remains the standard tool for research studies designed understand the effects of novel risk factors or treatments on peripheral artery conduit vessel function. Main recommendations for brachial artery flow-mediated dilation measurement are in Table 1.

**Table 1:** Recommendations for brachial artery flow-mediated dilation measurement.

**Patient instructions**
Fasting for at least 6 hours
No vasoactive medications for > 12 hours
No caffeine, cigarettes or nicotine for > 12 hours
No physical exercise for > 6 hours
Pre-menopausal women studied on day 1-7 of menstrual cycle
**Pre data acquisition**
Rest in supine position for at least 20 minutes (quiet and temperature-controlled room)
Blood pressure cuff positioned on forearm or upper arm
Brachial artery anatomical landmarks identification for serial studies
Linear array Doppler ultrasound machine > 7.5 MHz
**Data acquisition**
Ultrasound probe is positioned above the antecubital fossa in the longitudinal plane
Optimize image quality with clear identification of lumen interface Insonation angle ≤ 60
Baseline recording of diameter and velocity for 1 minute pre cuff inflation
Cuff inflation for 5 minutes at ≥ 50 mmHg above systolic pressure
Record post-cuff release diameter and hyperemia for 3 minutes

Adapted from Anderson & Phillips^[3]^.

### Microvascular Function

Regional blood flow is in large part controlled by tone in the smaller resistance vessels. Dysfunction of these vessels can lead to ischemia, particularly in the coronary circulation, as is the case in no-reflow or in chest pain syndromes and minimal CAD, syndrome X or microvascular angina.

#### Venous Occlusion Plethysmography

Prior to the introduction of the FMD technique, several groups reported on the assessment of changes in forearm blood flow in response to the infusion of intra-arterial vasoactive medications^[17]^. Plethysmography has been a technique to measure blood flow for more than 100 years and is based on the premise of measuring tissue (mainly muscle) blood flow reflected as a change in tissue volume. The standard approach is to use a calibrated mercury-in-silastic strain gauge placed around the forearm^[18]^. A BP cuff is used to isolate the hand and a proximal cuff is inflated to 40 mmHg to prevent venous outflow. Arterial inflow then results in expansion of the forearm with automated calibrated systems allowing the calculation of forearm blood flow in mL/min/100 mL tissue. Measurements are generally taken at rest and then after serial direct intra-brachial infusions of escalating doses of endothelium-dependent and independent agonists; Ach (or methacholine) and sodium nitroprusside have been most commonly used. The contralateral arm is used as a control in most laboratories. This provides important information about mechanisms responsible for attenuated responses seen with CV risk factors. Most CV risk factors have attenuated microvascular endothelial function^[19]^. Enzymatic inhibitors or receptor antagonists such as NO synthase inhibitors or adrenergic blockers can also be utilized.

### Reactive Hyperemia

Assessment of FMD typically involves the induction of a shear stress stimulus in the conduit brachial artery following ischemia induced by occlusion of the forearm vessels for 5 minutes. This shear stress is generated by dilation of the microvasculature, thereby producing increased velocity and flow (reactive hyperemia) in the conduit vessel. This can be measured by Doppler ultrasound after the release of the BP cuff and expressed as either peak velocity, the velocity time integral (VTI), forearm blood flow or systolic/diastolic ratio. Huang et al.^[20]^ demonstrated that hyperemic velocity was related to adverse events in subjects with peripheral vascular disease undergoing vascular surgery.

#### Arterial Biopsy and Isolated Resistance Vessel Function

Microvascular dysfunction is an important contributor to increased cardiometabolic risk. For example, impaired microvascular function can contribute to reduced insulin sensitivity, increased total peripheral resistance, and altered nutrient disposal^[21]^. In this context, studies of the isolated, resistance arteries defined by their internal diameter (50-200 µm) can be made by dissecting arterioles from tissue biopsies. These biopsies of the microcirculation have been made in the subcutaneous fat and muscle^[22]^. In addition, this technique to isolate arterioles from subcutaneous and visceral fat^[23]^, coronary arterioles discarded during surgery^[8]^. These preparations can involve the wire myograph^[24]^ or the isolated perfused arterial preparation with measurements using a video micrometer or an automated edge detector.

## ARTERIAL FUNCTION AND CARDIOVASCULAR DISEASE MANAGEMENT

Although recent studies have played important roles in the diagnosis and prognosis of endothelial assessment in individuals with CAD, there are several measures which are able to evaluate the endothelial function (EF; see above)^[3]^. In this context, Chan et al.^[25]^, in 2003 evaluated EF by FMD and endotheliumindependent dilations with 0.3 mg sublingually of nitroglycerin (NTG), in 152 CAD subjects. The results demonstrated that FMD and the FMD/NTG ratio were lower in subjects with cardiac events. This result supports the concept that both structural and functional properties of the vasculature are key co-determinants of CVD outcomes. In addition, the authors concluded that the assessment of EF is a predictor of future events, furthermore, the preserved carotid artery structure can mitigate the risk of future events related platelet formation, and the endothelial dysfunction reflects the propensity for atherosclerosis development in response to previous, longstanding exposure to risk factors.

In a recent study, Sugamata et al.^[26]^ assessed the EF in 923 CAD subjects, and were prospectively followed up for <8.5 years or until a coronary event - cardiac death, non-fatal myocardial infarction (MI) or unstable *angina pectoris* (uAP) requiring unplanned coronary revascularization. During the follow-up period, 116 events occurred (29 cardiac deaths, 46 non-fatal MIs and 41 cases uAPs). The results showed that FMD was independent predictor of secondary coronary events in patients with CAD. These findings indicate that FMD may contribute to be useful for risk stratification in patients with CAD.

In another study of patients with established CAD, Simova & Denchev^[27]^ showed similar results when evaluated in 198 subjects admitted to a hospital with angina and multiple risk factors divided into five groups according to the degree of CAD development. All patients had FMD and intima-media thickness (IMT) measured, 105 (53.03%) performed a treadmill test, and 146 (73.7%) underwent coronary arteriography (CAG). The results demonstrated that the patients with significant (≥ 50%) coronary artery stenosis had lower FMD and higher IMT values compared to patients without significant CAD. They concluded that endothelial evaluation presented important acceptance prognostic in CAD patients.

Furthermore, Kuvin et al.^[28]^, in 2001 evaluated endothelium-dependent vasomotion (EDV) with FMD as a predictor of the presence or absence of CAD as defined by exercise myocardial perfusion imaging (ExMPI) in 94 CAD subjects. The results demonstrated that subjects with CAD (n=23) had a lower FMD (6.3±0.7%) than those without CAD (10.5±0.6%). It was concluded that endothelial evaluation is highly sensitive for coronary disease.

Nevertheless, studies demonstrating the FMD technique in other CV diseases may indicate the diagnosis and prognosis role of this technique. Thereby, Wang et al.^[29]^ in 2009 in a follow-up, assessed the EF by FMD in 101 patients with acute MI and ST-segment elevation. The authors concluded that endothelial dysfunction significantly increased the risk of vascular events against to abnormal EF as a sensitive indicator of early development of atherosclerosis. In the same line of evidence, in 2010 Kazmierski et al.^[30]^ evaluated 75 men under 45 years of age, who were awaiting for elective coronary angiography in the hospital. The subjects were divided into two groups: study group (n=55) with obstructive coronary lesions and the control group (n=20) without lesions. The authors demonstrated a significant reduction of FMD for the study group when compared to control group (3.92±1.1 *vs.* 6.51±1.1). In this context, this finding demonstrated the relationship between the FMD and CV risk factors, estimating which risk factors have significant influence on EF. Additionally, these same authors investigated whether the assessment of EF by FMD can be useful for assessing the risk of CV disease in peri-menopausal women. The study involved 65 women with chest pain divided into a study group (coronary lesions, n=32) and a control group (without lesions, n=33). Atherosclerotic risk factors, early atherosclerotic remodeling by IMT and endothelial dysfunction measured by FMD were analyzed in all subjects. The results showed that IMT was significantly higher in the study group compared with controls (0.059±0.01 *vs.* 0.049 ±0.01 mm), moreover the FMD was significantly lower in the study group compared with controls (6.53±0.98 *vs.* 7.89 ±0.85). However, these results can be influenced by the presence of hypercholesterolemia and all components of the metabolic syndrome as well as by pharmacotherapy of these risk factors. The authors concluded that FMD can be an important diagnostic tool to assess CV risk^[31]^.

Therefore, in this context, in a meta-analysis by Inaba et al.^[32]^, FMD is closely related to the future of CV events, but further studies are needed to confirm the effectiveness of the use of FMD in CV disease management. In this sense, a study by Patti et al.^[33]^, evaluated the recurrence of stenosis in 136 patients with single-vessel CAD undergoing percutaneous coronary intervention (PCI) with stenting and at least 6 months of follow-up. All patients underwent FMD 30 days after PCI; and nitroglycerin-FMD were investigated before and after administration of sublingual NTG. The results showed that clinical in-stent restenosis was demonstrated in 20 (15%) patients, whereas 116 (85%) patients remained free of signs or symptoms of recurrent ischemia. Also, FMD was significantly impaired in patients with restenosis *versus* those without restenosis. The authors concluded that early evaluation of endothelial assessment may represent an important tool for predicting CV risk futures.

On the other hand, Frick et al.^[34]^ in 2005, assessed the prognostic value of FMD in a follow-up of 398 patients admitted to hospital with chest pain. Patients were divided into two groups according to the FMD median (7.6%), and after a mean follow-up of 39±12 months, CV events were documented. The results of the study demonstrated no difference in the number of CV events between groups. The authors concluded that IMT predicted late (up to 4 years) CV events, while the FMD technique is an important prognostic tool for CV event predictor.

Muiesan et al.^[35]^ evaluated 172 hypertension patients with the objective to evaluate the incidence of CV events during follow-up an evaluation of EF in the brachial artery. The results demonstrated that the CV events occurred in fewer patients with preserved FMD than in those with impaired FMD 23 (27%) *vs.* 9 (10.5%), with an incidence of 1.4 and 3.1 CV events per 100 patient-years. They concluded that uncomplicated hypertensive patients, followed up for an average period of 8 years, endothelial dysfunction, might identify patients at higher risk of CV events.

Considering others CV diseases, Meyer et al.^[36]^ assessed 75 CHF patients with a left ventricular ejection fraction (LVEF) ≤ 30%, which aimed to evaluate the predictive potency of impaired endothelium dependent in this population. The results showed that FMD significantly differed between event-free survivors or death in 75 CHF patients with LVEF ≤ 30%, FMD values were significantly impaired in CHF patients who reached the combined endpoint as compared with CHF survivors (5.4±5.1% *vs.* 11.2±7.4%). These finding may be the result of the impact of reduced EF in CHF. Therefore, reduced FMD may be an important tool for the composite physiological and prognostic assessment of patients with CHF.

## IMPACT OF EXERCISE PRESCRIPTION ON ARTERIAL FUNCTION

Physical exercise exerts beneficial effects on the human vascular system. Its acute and chronic impact has been targeted by many investigators over time^[9,37]^. The mechanism by which exercise results in improvement of arterial health is complex and is not fully understood. However, the effects of exercise on arterial function may be in part related to improvements in the endothelium function and health status.

Evidence suggests that exercise induces an up-regulation of endothelial NO synthase (eNOS) gene expression and vascular endothelial growth factor (VEGF)-induced angiogenesis; it also leads to a decrease in NO inactivation with augmented availability of antioxidants, such as superoxide dismutase (SOD) and glutathione peroxidase (GPx), and to an attenuation of nicotinamide adenine dinucleotide/nicotinamide adenine dinucleotide phosphate (NADH/NADPH) oxidase activity, leading to an increase in NO bioavailability^[9,37,38]^.

The shear stress effect on eNOS expression after exercise training was experimentally observed in animal models and in human studies^[39]^. Hambrecht et al.^[40]^ observed that a 4 weeks regular exercise training induced a 2-fold increase in eNOS mRNA expression and vascular protein content in stable CAD patients, suggesting that endothelial function improved possibly due to augmented eNOS phosphorylation. Maiorana et al.^[41]^, assessed conduit vessel endothelial function in diabetic patients using FMD of the brachial artery and demonstrated that 8 weeks of circuit training did not affect vascular function in healthy men. However, Clarkson et al.^[42]^ observed that FMD was enhanced following a 10 weeks program of daily aerobic and anaerobic exercise training in healthy young men. Other evidence from CAD patients showed that ET increases coronary blood flow through direct actions on the vasculature that improve endothelial function, enhancing coronary vasodilation^[43]^ and that even short-term ET could begin to reverse endothelial dysfunction in these patients, even though a longer duration is needed to restore endothelial function^[44]^.

Siasos et al.^[45]^ investigated the effects of continuous moderate and high intensity interval aerobic exercise on endothelial function and arterial stiffness in healthy subjects and demonstrated that both have favorable effects, suggesting a cardioprotective effect of both intensities of exercise. However, in another study, Franklin et al.^[46]^ showed that acute physical stress induced by resistance exercise reduces FMD in sedentary obese women, suggesting that this response to acute physical exertion may be an important marker for CV risk prediction in this population.

A wealth of studies has been published whose results support the beneficial effects of exercise training on arterial function. Evidence from subjects with endothelial dysfunction confirms that exercise training improves vasodilator function of resistance or conduit arteries^[44]^. However, findings from investigations on healthy asymptomatic subjects with presumably normal endothelial function are less consistent^[47]^. The acute effects of exercise on endothelium have been studied, but the number of studies is scarce and results are controversial. Another important aspect to be considered is that different exercise modalities are associated with different patterns of blood flow and arterial shear stress which may somehow impact on responses and adaptation of arterial function^[9]^. Besides, physiological adaptation may depend on exercise intensity, duration and frequency. Therefore, future studies to investigate the optimum exercise prescription in terms of modes, intensities and volume are needed. Table 2 summarizes the main exercise training programs studies assessing change in FMD CAD patients.

**Table 2:** Summary of exercise training studies assessing change in FMD in coronary arterial disease patients.

Study	Cohort	Study design	Exercise training	Outcome(s)
Van Craenenbroeck et al.^[60]^	Stable CAD	200 CAD randomized into 2 groups	12-wk of AIT vs. ACT on a bicycle, 3 times a week	Both training changed FMD% and peak VO_2_
Kim et al.^[59]^	Patients who received PCI due to ACS	32 nonrandomly divided into control (n=16) or CR (n=16) group	6-wk of 50-minute ACT, 3 times a week	peak VO_2_ and FMD (% and change) only for CR group
Currie et al.^[61]^	Stable CAD	22 CAD patients randomized into HIT (n=11) or ACT (n=11) based on pretraining FMD	12-wk of ACT (30-50 min of cycling at 58%WR vs. HIT (10 1-min intervals at 89% separated by 1-min intervals at 10% WR)	Both training changed FMD% and peak VO_2_
Luk et al.^[58]^	Stable CAD	64 randomized into control (32) and exercise training program	8-wk of ACT program	FMD (1.84%) and exercise capacity (2 METS) only in ACT group
Walsh et al.^[62]^	Stable CAD	20 CAD patients randomized in 2 groups (trained vs. controls)	8 wk of combined aerobic (70% MHR) + resistance circuit training (55-65% MR)	FMD (%) and glyceryl trinitrate GTN-mediated dilation only in trained group

ACT=aerobic continuous training; ACS=acute coronary syndrome; AIT=aerobic interval training; CAD=coronary arterial disease; CR=cardiac rehabilitation; GTN=glyceryl trinitrate; HIT=high intensity training; MET=metabolic equivalent; MHR=maximal heart rate; MR=maximal repetition; PCI=percutaneous coronary intervention; VO_2_=oxygen uptake; WR=work rate.

## EXERCISE, ARTERIAL FUNCTION AND CARDIAC REHABILITATION

Exercise-based cardiac rehabilitation (CR) is widely recognized as a non-pharmacological adjunct able to reduce all-cause and cardiac mortality and the incidence of acute cardiac events, to modify risk factors associated with CVD and to mitigate CV disease progression^[37]^. Exercise training positive effects on CV risk exposure are partially due to its direct effects on the vessel wall^[48]^. Exercise induces increases in blood flow and shear stress, which are recognized as important physiological stimuli for arterial function improvement^[37]^.

Long-term vasculature adaptations determined by physical exercise result in an improvement of systemic arterial vascular function not just in healthy individuals, but also in patients affected by CVD. Arterial health can be compromised in various pathological conditions affecting the cardiovascular system and in the presence of CVD risk factors. Evidence shows that exercise is beneficial for patients suffering from a variety of chronic diseases, including hypertension (HTN), coronary heart disease (CHD), heart failure (HF) (reviewed above), obesity and insulin resistance^[9]^.

Higashi et al.^[38]^ investigated FMD in HTN subjects and showed that moderate intensity aerobic exercise significantly improved endothelial health in this population. Collier et al.^[49]^ showed that arterial function was improved in HTN patients following 4 weeks of either aerobic or resistance training. Most of the studies that investigated ET effects on endothelial function reported correlations with improved insulin sensitivity and other insulin mediated metabolic functions^[9]^. Hambrecht et al.^[44]^ studied the cardioprotective effects of ET in CAD patients and showed that 4 weeks of ET on a cycle ergometer improved endothelium-dependent vasodilation both in epicardial coronary vessels and in resistance vessels.

Collectively, evidence suggests that arterial function is amenable to improvement in subjects at known or increased cardiovascular disease risk who undertake physical exercise training. In the sections that follow, we will present existing evidence related to how exercise training can be applied for primary and secondary CVD prevention.

### a. Primary Prevention

Evidence suggests that lifestyle modification via regular exercise has significant preventive effects against CVD by reducing multiple risk factors; on the other hand, physical inactivity is largely associated with CVD development. One of the factors that contribute to the genesis of CVD^[37]^.

Impaired endothelium-dependent vasodilation appears to be the earliest event of atherosclerosis and is therefore considered the focus of strategies for CVD prevention. Moreover, endothelial dysfunction has been extensively associated with most of the established CV risk factors (*e.g*. dyslipidaemia, hypertension, smoking, and diabetes mellitus). Experimental findings demonstrate that exercise training is able to prevent and improve endothelial dysfunction. Furthermore, management of CV risk factors also tends to improve or restore endothelial function^[9,50]^.

Jacomini et al.^[51]^ studied the influence of the training status on blood pressure in older women and showed that better performance in a Fitness Battery Test and higher maximal oxygen uptake were related to lower levels of oxidative stress and protein damage. Moreover, the authors observed that a better training status is related to a higher concentration of nitrite and nitrate, a decrease of which was shown to be correlated with increasing numbers of CV risk factors and endothelial dysfunction.

Regular aerobic exercise was also associated with improvements in conduit artery and microvessel FMD, following an acute strenuous physical exertion in overweight and obese individuals. Since acute stress to the vascular endothelium is linked to higher incidence of CVD, physical exercise practice appears to be fundamental to protect against vascular dysfunction^[50]^.

The acute effects of continuous moderate intensity aerobic exercise (CAE) and high intensity interval aerobic exercise (HIAE) on endothelial function and arterial stiffness were investigated by Siasos et al.^[45]^ in healthy individuals. Participants performed an isocaloric protocol on a cycle ergometer and presented significantly improved FMD after both protocols. The authors suggested a cardioprotective effect of exercise on the progression of atherosclerosis.

### b. Secondary Prevention

It is clearly recognized that physical inactivity is related to increased risk for morbidity and mortality and contributes to health care costs, particularly in patients with CVD^[52]^. Therefore, Schmidt et al.^[53]^ have recently focused on evaluating the mechanisms responsible for the systemic effects of physical exercise on secondary CV prevention.

The potential beneficial effects of exercise are multifaceted. In the context of vascular function, physical exercise induces upregulation of eNOS expression and activity, which has been associated to consequent endothelium-dependent vasodilatation^[54]^. This phenomenon is linked to consequent peripheral neovascularization including arterial function and angiogenesis. In addition, the vascular system is expanded in size and number of microvessels in patients with CAD^[55]^.

Attention has focused on noninvasive tools to measure of arterial function in CAD patients. Based on the ability of the endothelium to acutely dilate in response to an increase in blood flow, the FMD has been constituted as an important index of endothelial function predictive of future CV events in chronic arterial disease^[56]^. In addition, impaired endothelial function has been shown to limit exercise performance in CAD patients^[57]^, even as a lower level of habitual PAL is associated with impaired FMD in these patients^[58]^. FMD also have demonstrated to be a reproducible measurement for short- and medium-term assessment of pharmacological and nonpharmacological interventions, such as exercise training. However, different modalities of physical training program, training intensity duration, as well as volume of exercise training, can influence on endothelial function responses in coronary disease patients.

For instance, Kin et al.^[59]^ applied a protocol based on six-week and three times a week of aerobic exercise training program in a group of patients who were hospitalized due to acute coronary syndrome. The intensity of exercise training program consisted of 40%-85% of the heart rate reserve and the total duration were 30 minutes, intercalating rest periods with exercise on a cycle and treadmill. They observed a significant improvement of FMD change after 6 weeks, however, a none significant difference was observed when compared to controls. However, 12 weeks of both aerobic interval training and continuous exercise training on cycle could significantly improve aerobic capacity (as expressed by VO_2_ peak), markers of endothelial integrity and FMD percentage in a large population of CAD patients^[60]^.

On the other hand, Currie et al.^[61]^ contrasted 12 week of low-volume high-intensity interval exercise training *versus* higher volume, moderate intensity endurance exercise. Both training program significantly increased relative FMD post training in a similar way, with no differences between groups. These results taken together show that longer protocols (12 weeks) seem to demonstrate better responses, and that, in addition, continuous or interval training may be both important training strategies to improve endothelial function in patients with CAD. In addition, Walsh et al.^[62]^ tested the effects of resistance associated to aerobic exercise training program was applied during 8 weeks, three times a week. The authors observed that combined exercise training improved FMD percentage, but not the responsiveness of endothelium-independent vasodilation, measured by administration of glyceryl trinitrate.

Although there is already a certain body of knowledge about the potential beneficial effects of physical exercise on endothelial function in patients with CAD, there is still a need for future studies to evaluate long-term effects of physical training in these patients. In addition, there is still no evidence for the effects of high versus low continuous intensity training, as well as the effects of resistance training and strength training in these patients. Thus, new studies in order to evaluate these existing gaps still become necessary in future studies.

### c. Peripheral Arterial Disease

Peripheral arterial disease (PAD) affects eight million men and women in the USA, however, in Brazil, data related to the prevalence of PAD and its risk factors are scarce and restricted to specific populations. Endothelial function is impaired in individuals with PAD compared to individuals without PAD^[63]^. On the other hand, higher levels of physical activity during daily life are associated significantly and independently with better brachial artery FMD among individuals with PAD.

In this context, Januszek et al.^[64]^ showed that 12 weeks of supervised, intermittent treadmill walking until induce claudication, three times a week, prolonged the asymptomatic walking distance and improved brachial artery FMD in 43% after program. Another study that tested the hypothesis of potential effects of endothelial function in PAD patients that underwent to a supervised treadmill training program of 12 weeks found that brachial artery FMD values increased by 45% after program and the patients with better walking abilities at baseline derived greater clinical and metabolic benefits^[65]^. However, a recent meta-analysis study observed that exercise training, compared with medical care, significantly improved cardiorespiratory fitness, pain-free and total flat-ground walking distances, as well as graded treadmill performance in PAD, but not ankle-brachial index and FMD in PAD patients^[66]^.

In conclusion, to date, the effects of resistance and strength training on FMD in PAD patients. New studies with the objective of testing the potential effects of resistance exercise protocols can be conducted in the future.

## ARTERIAL FUNCTION, REHABILITATION AND STROKE

Considering that endothelial dysfunction plays a role in the development and progression of atherosclerosis, ischemic stroke is also strongly associated with endothelial dysfunction. In fact, patients with acute ischemic stroke have impaired FMD in comparison with control subjects, FMD is negatively related to stroke severity, and impaired FMD is also associated with poor outcome in these patients^[67]^. In addition, FMD ≤ 4.5% was an independent predictor of a new-onset vascular event after stroke, with lower event-free survival rates within 48 months after a first ischemic stroke^[68]^.

It is well established that physical activity, as a modifiable risk factor, has beneficial effects on cardiovascular health^[37]^. Improvement on endothelial function and neovascularization related to regular exercise training contribute to these results, therefore, it is reasonable to predict the importance of exercise on prevention of cerebrovascular disease^[53]^. The Cardiovascular Health Study found that greater levels of physical activity were associated to lower incidence of stroke in a population of 4207 US men and women with age ≥ 65 years followed for 10 years^[69]^. The Women's Health Study also demonstrated that leisure time physical activity was associated to lower stroke risk in healthy women ≥ 45 years followed for 11.9 years, in average^[70]^.

There are few studies related to endothelial function after rehabilitation in stroke patients. Billinger et al.^[71]^ applied 8 weeks of aerobic exercise three times per week in 9 subacute stroke patients. They evaluated brachial artery FMD in both arms (stroke-affected and non-affected limbs) at baseline, postintervention and 1 month follow-up. The authors found lower FMD in the affected arm at baseline and at post-intervention measurements, with improvement on vascular health after treatment with sustained improvement on follow-up. Takatori et al.^[72]^ performed a randomized controlled study investigating the effects of intensive rehabilitation for 12 weeks, twice a week, on physical and arterial function of chronic stroke survivors patients. The authors found a significant improvement on arterial function (cardio-ankle vascular index and ankle-brachial pressure index) after the rehabilitation period on experimental group in comparison with control group, despite of no changes on physical function.

## ARTERIAL FUNCTION AND CARDIOVASCULAR SURGERY

The diagnostic value of microvascular and conduit artery endothelial dysfunction has increasingly been investigated in the cardiovascular population^[73]^. Flow-mediated dilation, quantified by noninvasive methods such as brachial artery reactivity testing (BART), is impaired in patients with CAD^[73]^, providing improved sensitivity and negative predictive value for the diagnosis of CAD. Cardiovascular risk factors predispose to perioperative morbidity and mortality, with evidence that patients with microvascular dysfunction undergoing cardiovascular interventions are at increased risk for postoperative complications^[73]^.

In this context, Patti et al.^[33]^ included in a prospective study 136 patients with single-vessel CAD undergoing percutaneous coronary intervention (PCI) with stenting and at least 6 months of follow-up. The authors verified whether early measurement of brachial artery FMD after coronary stenting could predict occurrence of in-stent restenosis. All patients underwent ultrasound detection of brachial artery reactivity 30 days after PCI. FMD was significantly impaired in patients with restenosis versus those without restenosis (percent diameter variation 4.6 ± 5.8% *versus* 9.5 ± 6.6%, *P*=0.002); moreover, 4% of patients with FMD ≥ 7% (median value) developed in-stent restenosis *versus* 28% of those with FMD < 7% (*P*=0.0001). The results this study indicating that impaired FMD independently predicts occurrence of in-stent restenosis in patients undergoing PCI and may represent a useful screening tool to stratify patients according to future risk of restenosis.

Cardiopulmonary bypass (CPB) exerts several deleterious effects in patients undergoing myocardial revascularization, including inflammatory pathways. Most of these can be related to an endothelial insult leading to endothelial dysfunction. Sangalli et al.^[74]^ using FMD of the brachial artery assessed 29 patients undergoing elective myocardial revascularization. Ten patients receiving continuous-flow CPB, 10 receiving pulsatile-flow CPB, and 9 scheduled for beating-heart revascularization were studied. Patients were studied at baseline (after induction of general anesthesia), after CPB upon intensive care unit (ICU) admission after surgery, and on the first postoperative day before discharge from the ICU (on average, 24 hours after CPB discontinuation). The continuous-flow CPB group demonstrated a significant reduction in FMD after CPB (12.8±9.7% *vs.* 1.6±1.5%, *P*<0.01), which lasted up to the first postoperative day (5.9%±4.1%). On the other hand, FMD did not change in the pulsatile-flow group (12.5±10.5%, 11.0±7.2%, and 16.6±11.7%, respectively). FMD also was unaffected in the beating-heart group, thus suggesting a direct effect of CPB itself on endothelial function. In conclusion, in these patients, continuous-flow CPB markedly impaired endothelial function, although this was not the case with pulsatile-flow CPB group.

Gokce et al.^[75]^ preoperatively examined brachial artery vasodilation using ultrasound in 187 patients undergoing a vascular operation, including carotid endarterectomy (47 patients), femoral-popliteal or other peripheral bypass (100 patients), aortic aneurysm repair (24 patients), and limb amputation (16 patients). Patients were prospectively followed for 30 days after surgery. Forty-five patients had a postoperative event, including cardiac death (3), myocardial infarction (12), unstable angina/ischemic ventricular fibrillation (2), stroke (3), or elevated troponin I, reflecting myocardial necrosis (25). Preoperative endothelium dependent FMD was significantly lower in patients with an event (4.9±3.1%) than in those without an event (7.3±5%; *P*=0.001), whereas endothelium-independent vasodilation to nitroglycerin was similar in both groups. In a Cox proportional-hazards model, the independent predictors of events were age (*P*=0.001), renal insufficiency (*P*=0.03), non-carotid surgery (*P*=0.05), and lower brachial artery FMD (*P*=0.007). In this study, impaired brachial artery endothelial function was independently predicts postoperative cardiac events, which supports a role for endothelial dysfunction in the pathogenesis of cardiovascular disease.

The primary endpoint of this study, Schier et al.^[76]^ investigated whether preoperative BART-derived variables would predict postoperative complications, not restricted to cardiovascular events but inclusive of all postoperative complications that commonly occur after major thoracic surgery. The authors investigated 63 patients using BART-derived variables, including FMD, in preoperative risk stratification for major thoracic surgery. Patients in the low FMD group experienced more postoperative complications when compared high FMD: 54% *versus* 30% had one or more adverse postoperative event, and 11% *versus* 0 had three or more adverse postoperative events (*P*<0.001), respectively. The authors concluded that preoperative microvascular dysfunction can be identified in patients at increased risk for postoperative complications.

## CONCLUSION

The understanding of the vascular regulation of blood flow and perfusion in the peripheral circulation has grown dramatically in the past 3 decades. The endothelium plays a crucial role in regulating blood vessel function and is an important prognostic indicator of arterial function and cardiovascular risk. Alterations in arterial function are critical in the pathogenesis of cardiovascular disease including the risk factors that lead up to overt CAD and CV surgery. In addition, rehabilitation strategies in the context of primary and secondary prevention for CV disease (hypertension, obesity, stroke, MI) and following surgery have important positive impacts on nitric oxide and hence conduit artery endothelial function. Future focus should remain on the optimal mode and intensity of such rehabilitation strategies for the benefit of endothelial function and lowering CV risk and disease.

**Table:** 

Authors' roles & responsibilities	
SAP	Conception and design study; manuscript redaction or critical review of its content; final manuscript approval
DKA	Conception and design study; manuscript redaction or critical review of its content; final manuscript approval
RGM	Conception and design study; manuscript redaction or critical review of its content; final manuscript approval
FRC	Conception and design study; manuscript redaction or critical review of its content; final manuscript approval
RC	Conception and design study; manuscript redaction or critical review of its content; final manuscript approval
RBJ	Conception and design study; manuscript redaction or critical review of its content; final manuscript approval
RA	Conception and design study; manuscript redaction or critical review of its content; final manuscript approval
ABS	Conception and design study; manuscript redaction or critical review of its content; final manuscript approval
